# 2444 Development of an instrument to identify factors influencing point of care recruitment in primary care settings: A pilot study at University of Utah Health

**DOI:** 10.1017/cts.2018.162

**Published:** 2018-11-21

**Authors:** Teresa Taft, Charlene Weir, Heidi Kramer, Julio Facelli

**Affiliations:** The University of Utah School of Medicine

## Abstract

OBJECTIVES/SPECIFIC AIMS: Electronic health records have become the fulcrum for efforts by institutions to reduce errors, improve safety, reduce cost, and improve compliance with recommended guidelines. In recent times they are also being considered as a potential game changer for improving patient recruitment for clinical trials (CT). Although the use of CDS for clinical care is partially understood, its use for CT patient identification and recruitment is young and a great deal of experimental and theoretical research is needed in this area to optimize the use of CDS tools that personalize patient care by identifying relevant clinical trials and other research interventions. The use of CDS tools for CT recruitment offers a great deal of possibilities, but some initial usage has been disappointing. This may not be surprising because, while the implementation of these interventions is somewhat simple, ensuring that they are embedded into the right point of the care providers workflow is highly complex and may affect many actors in a clinical care setting, including patients, nurses, physicians, clinical coordinators, and investigators. Overcoming the challenges of alerting providers regarding their patient’s eligibility for clinical trials is an important and difficult challenge. Translating that effort into effective recruitment will require understanding of the psychological and workflow barriers and facilitators for how providers respond to automated alerts requesting patient referrals. Evidence from using CDS for clinical care that shows alerts become increasingly ignored over time or with more exposure (1, 2). The features, timing, and method of these alerts are important usability factors that may influence effectiveness of the referral process. Focus group methods capture the shared perspectives of a phenomenon and have been shown to be an effective method for identifying perceptions, attitudes, information needs, and other human factors effecting workflow (3, 4). Our objective was to develop a generalizable method for measuring physician and clinic level factors defining a successful point of care recruitment program in an outpatient care setting. To achieve this we attempted to (a) Characterize provider’s attitudes regarding CTs referrals and research. (b) Identify perceived workflow strategies and facilitators relevant to CT recruitment in primary care. (c) Develop and test a pilot instrument. METHODS/STUDY POPULATION: The methods had 3 phases: focus groups, development of item pool, and tool development. Focus group topics were developed by 4 experienced investigators, with training in biomedical informatics, cognitive psychology, human factors, and workflow analysis, based upon a knowledge of the literature. A script was developed and the methods were piloted with a group of 4 clinicians. In all, 16 primary care providers, 5 clinic directors, and 6 staff supervisors participated in 6 focus groups, with an average of 5 participants each, to discuss clinical trial recruitment at the point of care. Focus groups were conducted by the development team. Audio recording were content coded and analyzed to identify themes by consensus of 3 authors. Item Pool generation involved extracting items identified in the focus group analysis, selecting a subset deemed most interesting based on knowledge of the recruitment literature and iteratively writing and refining questions. Instrument development consisted of piloting an initial 7-item questionnaire with a local primary provider sample. Questions were correlated with the item pool and limited to reduce provider burden, based on those that the study team deemed most applicable to information technology supported recruitment. Descriptive statistical analysis was performed on the pilot survey results. An online survey was developed based on the findings of the focus groups and emailed to 127 primary care providers who were invited to participate. In total, 36 questionnaires were completed. This study was approved by the University of Utah Institutional Review Board. RESULTS/ANTICIPATED RESULTS: The results section is organized into 3 sections: (a) Focus groups, (b) Item generation; and (c) Questionnaire pilot. (I) (1) Focus Groups. Themes identified through a qualitative review are presented below with illustrative comments of participants. The diversity of attitudes and willingness to support clinical trial recruitment varied so substantially that no single pattern emerged. Attitudes ranged from enthusiastic support, to interest in some trials to disinterest or distrust in trials in general. Compensation for time spent, which could be monetary, informational, or through professional recognition; and provider relationship with the study team or pre-selection of specific trials by a clinic oversight committee, and importance to providers practice positively affected willingness to help recruit. “I would love to get people into clinical trials as much as possible... If it works for them you are going to help a whole lot of other people.” If we felt like we have done every possible thing that was already established as evidence-based and it didn’t work out, then we would consider the trials. I think that studies are more beneficial for specific specialists... There might be a whole slew of things that I never deal with or don’t care about because it’s not prevalent for my patient population. Local and reputable... A long distance someone asking to do something is just not the same as someone in the trenches with you. The bottom line is how much work is involved at our end and if there is going to be any compensation for that. I think also the providers would like have feedback on what they referred them to. And how did it go? So did we pick the right patient? ... It helps us to know, did they even sign up for the study? Getting your name on a research paper would be nice too. Lack of information regarding trials reduced support for recruitment of patients. Providers stated that they do not know how to quickly find information about studies, nor do they have time to find the information, and therefore cannot efficiently council patients regarding trial participation. Notifications regarding clinical trials that were deemed to be important included: Trial coordinator intention to recruit patients, enrollment of a patient in a clinical drug trial, trial progress and result updates, and reports of effectiveness of provider recruitment efforts. Perceived information needs regarding trials that providers are referring patients to included: trial purpose, design, benefits and risks, potential side effects, intervention details, medication class (mechanism of action), drug interactions with study drug, study timeline, coordinator contact information, link to print off patient handouts, enrollment instructions, and a link to study website. (2) It’s just we don’t know any of the information ... and it can’t take any of our time. ... I don’t have time to research it. Sometimes the patients ask me questions about it and I would like to be in a position where I have some information about it before I am asked. It would be nice to be notified if they [my patients] are enrolled in the trial, when it turns into actual recruitment. I do like to know if they’re in [a trial] so that when they come in for problems, I at least know that they might be on a study medication so I can be safe. I’ll get an ER message, “The patient got admitted. There blood pressure’s, you know, tanked, because they’re on a study drug I didn’t know anything about.” if there’s certain side effects that I need to be watching out for. It would also be good to have a contact person from the study in case we need to notify them of. “this person’s possible having an adverse event. Look into it more.” (3) Provider burden associated with patient recruitment appeared to be a deterrent. These burdens included adding to the providers task list, increasing the time required to complete a visit, and usurpation of control over the patients care plan with the associated effect on provider quality scores. We don’t have time. I mean, we don’t even take a lunch break. I have 15 minutes and now this is taking this many minutes away from my 15 minutes. I am just sick of extra work. We already have so much extra work. It’s just more stuff to do. We are maxed out on stuff to do. Right now, part of our compensation depends on having our patients A1Cs controlled. And so if we’re taking a chance that maybe they’re getting a medicine, maybe they’re not, maybe it’ll help, maybe it won’t, its gonna further delay our ability to get paid. Cause they’re like “I’m not going to let you go mess up my patient and I’m going to have to deal with the consequences is kind of the way they think. If you’re going to put the patient in a study, being able drop them from our registry so we don’t get penalized for a negative outcome [is important]. (4) Patient’s needs were a priority among factors influencing likelihood to help recruitment patients. Providers considered perceived benefit or risk to the patient, such as additional healthcare services, increased monitoring, financial assistance, or access to new treatments when other options have been ineffective, important; as well as continuance of established care that has proven effective, and ethical recruitment that addresses language and mental health to ensure that patients can make decisions regarding study participation. If there’s something great that’s gonna benefit a patient, I would definitely wanna know about it to give them that option. You know that’s what we wanna try to do is make our patients better. Someone who is really well controlled and doing well, I would not tend to put them toward the study. Just keep going with what’s working right now. Sometimes there’s financial incentives for them to participate, so you know, if its a good fit its easy to at least offer that to the patient. They get treatment maybe that they can’t afford. You don’t want to be seen as somebody who's forcing a patient... if their provider is telling them this is a good idea you are more likely to get your patient to do it. I think they have to understand what a clinical trial is, first of all, in that it’s a trial. Right? We’re trying to figure out if a certain treatment is good or not. It may not work. It may work. With many patients, they don’t only have medical problems, but significant mental illness that sometimes interferes a lot with just our treatment of them here for their clinical problems. And so, that probably would interfere with someone’s ability to understand and consent to a trial. And the patients have the right to make that choice. I don’t need to be—I don’t mind influencing them on things I know about, I think are invaluable, but I don’t need to be a barrier to them. (5) Perceived responsibility in trial recruitment varied substantially, from no involvement at all, to prescreening, counseling, or recruiting patients. Some providers felt that they should have the right to say “no” to recruitment of their patients while others believed prescreening was an unnecessary burden, outside of their role as a primary care provider. if someone prescreens and thinks its appropriate and gives me that judgment call to say, do you think it would be a good fit? I think one of them, they sent, and I said, Oh, I don’t think it would be a good fit because of this...So that would be fine. I don’t think I need to be a gatekeeper for studies. I mean, if there’s people that qualify for a study, and there’s a great study that’s been approved, and they can recruit them without me knowing, that doesn’t bother me in the slightest. I liked how it was—I could do a simple referral ... someone else figured out the qualifications. if we knew of ongoing studies and if we thought a certain patient may qualify for a certain study, we just contact the coordinator, and then they just take care of the rest. I think that appropriate ... from our perspective, would be, “Are you interested?” “This is the number for a person who can sit with you, talk with you about a trial, tell you everything about it, answer your questions, and then you can make a decision.” I’m not going to let you go mess up my patient and I’m going to have to deal with the consequences. (6) A clinic-implementation approach that systemizes workflow, limits the number of trials providers are asked to recruit for, and minimizes provider time burden is needed. Suggested methods for informing providers of patient clinical trial eligibility included: email, alerts, in-basket messages, texts, phone-calls, and in-person contact. People are so sick of change, change, change, change ... if there’s no stability whatsoever, then people get frustrated and start to burn out. Having my staff remember how to do it correctly and I remember what studies we have going ... it becomes somewhat of a burden... it’s hard for us to remember as we are flying through our day. There just needs to be a clear understanding with those roles... Who does the patient call? We don’t want to look like we don’t know what we are doing. There probably should be a selection committee put together from various people who have stakes in the community, at least who can say, “This would be applicable for xx clinic.” (7) Provider Suggestions Providers had multiple suggestions regarding notification methods. (II) Development of item pool and construction of questionnaire The specific items were constructed from literature review on physician’s attitudes and results from the focus group. The overarching concern was on readability, brief questionnaire size, and relevance. A large item were constructed and then reduced through piloting. (III) Questionnaire Pilot Results: The 7-item pilot questionnaire was completed by 36 physicians (28% response rate). In this section, we report the empirical results. DISCUSSION/SIGNIFICANCE OF IMPACT: Discussion Relevance of Methods. Overall, the described methods for determining components for a recruitment program in primary care shows early promise. The focus groups that consisted of providers, staff and administrators resulted in insights as to workflows, attitudes, and clinical processes. These insights significantly varied across clinics. This variation supported the need for an individualized clinic-based approach that will meet local needs. During the course of the study, participants were willing to participate in all activities (although some requested payment). We were able to conduct the focus groups as scheduled and obtained the desired input. The analysis of the focus group transcripts was performed using iterative discussions and did not needed any special adaptation for this area of study. The pilot survey response rate was within the expected for this type of study. Focus groups can rapidly provide rich information regarding attitudes and other factors affecting provider participation at the point of care. However, findings from focus groups must always be confirmed through larger studies. It is important to keep the focus groups small and to hold multiple focus groups to offset the more vocal participants that may influence comments of others. This study shows that using our 3-step approach it is possible to gather important information on clinician’s and staff perceptions and needs to participate in point of care patient recruitment for CT. The focus groups also provide an important step for survey construction. Designing surveys empirically requires multiple validation efforts, which will be conducted in the future. However, we can draw preliminary conclusions from the results of the pilot study which are quite informative and they are discussed below. Near future work will be to expand the response rate through additional local survey and conduct formal psychometric testing and validation both locally and nationally. A final validation will be proposed through the CTSA consortiums. Variation in responses. There was a lack of normal curves in our survey results. This points to the need to target education and recruitment efforts by provider type (with similar perspectives). Identification of these types would be useful. Some specific points regarding variability that should be considered in program design. Preferences for trail recruitment methods. Many trial recruitment notification methods have the potential to be successful when used judiciously and done well, particularly if the trial coordinator/provider relationship is supported by reciprocal benefits to the provider. Consistency in workflow within seems paramount to success. Providers can pull some notifications at a time they choose, while other notifications interrupt and must be used sparingly. Some allow review of multiple patients at the same time, and some foster easy access to the patient’s medical record. Conclusions. The authors recommend that recruitment HIT be customizable at the clinic and provider level by responsibility and interest to allow selection of level of information, delivery method, that is, email, text, in-basket, alert, dashboard, mail; frequency of notification, and an opt out feature. These customizable options will allow for better support of clinic workflow or goals. There is the potential with machine learning technology to monitor provider interactions with trial notifications and for the system to automatically make adjustments to the method and level that best supports each physician. Limitations: The major limitation is the focus on one site only and one delivery system (university based). The low response makes generalization difficult. Efforts to improve the rate are underway. Many populations are under-represented in Utah. Full psychometric analysis was not conducted but will part of the final project.

